# Endoscopic ultrasound-guided palliative enterocolostomy via lumen-apposing metal stent in the setting of ascites and Roux-en-Y gastric bypass

**DOI:** 10.1055/a-2223-4157

**Published:** 2024-01-09

**Authors:** Jason DuBroff, Daryl Ramai, John D. Morris

**Affiliations:** 1 14434 Division of Gastroenterology, Hepatology, and Nutrition, University of Utah Health, Salt Lake City, United States

Endoscopic ultrasound-guided enterocolostomy (EUS-EC) has recently been shown to be a novel technique for palliation of malignant, distal, small-bowel obstruction 1 . We present a case in which EUS-EC was successfully created via a lumen-apposing metal stent (LAMS) as an alternative to standard interventions for palliative decompression.

A 44-year-old woman with previous history of Roux-en-Y gastric bypass and recently diagnosed stage IV adenocarcinoma of unknown primary was admitted for small-bowel obstruction secondary to peritoneal carcinomatosis ( Video 1 ). The patient was a poor candidate for standard palliative interventions (i.e. decompressive gastrostomy) owing to peritoneal disease, ascites, and Roux-en-Y gastric bypass surgical anatomy.

A patient with Roux-en-Y gastric bypass and metastatic adenocarcinoma of unknown primary complicated by malignant ascites was admitted for distal small-bowel obstruction. Endoscopic ultrasound-guided enterocolostomy was successfully performed after therapeutic paracentesis.Video 1

Computed tomography on admission showed the descending colon to be in proximity to the distended small bowel ( Fig. 1 ). This was confirmed with lower EUS; however, the initial window was suboptimal due to the presence of ascites. The decision was made to perform a paracentesis the day of repeat procedure. Repeat lower EUS then demonstrated large and proximal small bowel in close apposition. After identifying the target small bowel, a 19-gauge needle was used for transluminal injection of contrast and a small-bowel enterogram was obtained. A cautery-enhanced LAMS (20×10 mm AXIOS Stent with Electrocautery Enhanced Delivery System; Boston Scientific, Marlborough, Massachusetts, USA) was deployed under EUS and fluoroscopic guidance for the creation of an enterocolonic anastomosis ( Fig. 2 ). The stent was appropriately positioned and small-bowel succus was observed flowing from the stent. The patient was treated with broad-spectrum antibiotics during the periprocedural period.

**Fig. 1:**
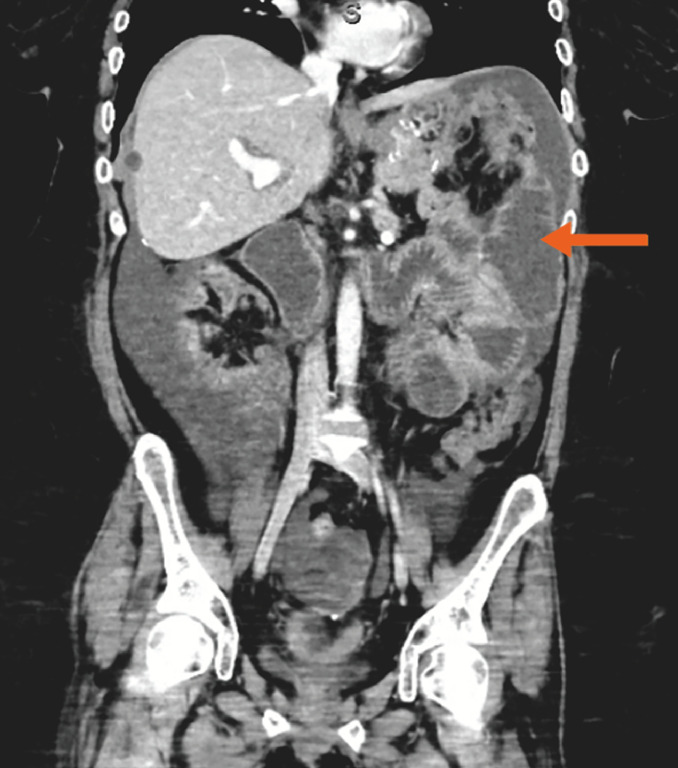
Descending colon in proximity to the jejunum (arrow).

**Fig. 2:**
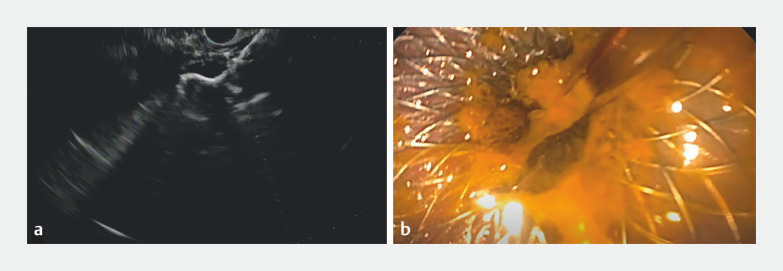
Deployment of the lumen-apposing metal stent (AXIOS; Boston Scientific, Marlborough, Massachusetts, USA). **a** Endoscopic ultrasound deployment of the stent. **b** Endoscopic view of the deployed stent.

The patient experienced immediate relief of obstructive symptoms. Appropriate positioning of the stent was confirmed by computed tomography and her diet slowly advanced to full liquids ( Fig. 3 ).

**Fig. 3:**
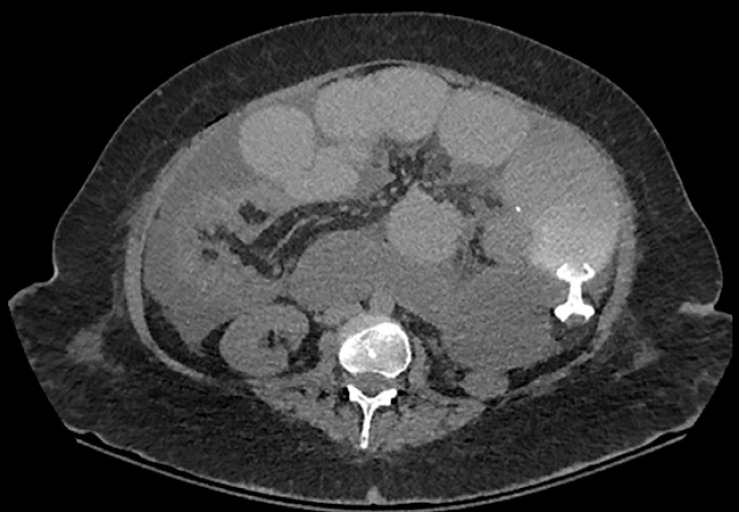
Computer tomography scan showing correct positioning of the lumen-apposing metal stent (AXIOS; Boston Scientific, Marlborough, Massachusetts, USA).

EUS-EC is an alternative intervention for the palliation of malignant small-bowel obstruction in selected patients, offering the possibility of symptomatic relief and oral nutrition.

Endoscopy_UCTN_Code_TTT_1AS_2AZ

## Notes

Endoscopy E-Videos https://eref.thieme.de/e-videos
*E-Videos* is an open access online section of the journal *Endoscopy* , reporting on interesting cases and new techniques in gastroenterological endoscopy. All papers include a high-quality video and are published with a Creative Commons CC-BY license. Endoscopy E-Videos qualify for HINARI discounts and waivers and eligibility is automatically checked during the submission process. We grant 100% waivers to articles whose corresponding authors are based in Group A countries and 50% waivers to those who are based in Group B countries as classified by Research4Life (see: https://www.research4life.org/access/eligibility/ ). This section has its own submission website at https://mc.manuscriptcentral.com/e-videos .
